# Genetic structure and breeding system in a social wasp and its social parasite

**DOI:** 10.1186/1471-2148-8-239

**Published:** 2008-08-20

**Authors:** Eric A Hoffman, Jennifer L Kovacs, Michael AD Goodisman

**Affiliations:** 1 School of Biology, Georgia Institute of Technology, 310 Ferst Drive, Atlanta, GA 30332, USA; 2 Department of Biology, University of Central Florida, 4000 Central Florida Blvd., Orlando, Florida 32816, USA

## Abstract

**Background:**

Social insects dominate ecological communities because of their sophisticated group behaviors. However, the intricate behaviors of social insects may be exploited by social parasites, which manipulate insect societies for their own benefit. Interactions between social parasites and their hosts lead to unusual coevolutionary dynamics that ultimately affect the breeding systems and population structures of both species. This study represents one of the first attempts to understand the population and colony genetic structure of a parasite and its host in a social wasp system.

**Results:**

We used DNA microsatellite markers to investigate gene flow, genetic variation, and mating behavior of the facultative social parasite *Vespula squamosa *and its primary host, *V. maculifrons*. Our analyses of genetic variability uncovered that both species possessed similar amounts of genetic variation and failed to show genetic structure over the sampling area. Our analysis of mating system of *V. maculifrons *and *V. squamosa *revealed high levels of polyandry and no evidence for inbreeding in the two species. Moreover, we found no significant differences between estimates of worker relatedness in this study and a previous investigation conducted over two decades ago, suggesting that the selective pressures operating on queen mate number have remained constant. Finally, the distribution of queen mate number in both species deviated from simple expectations suggesting that mate number may be under stabilizing selection.

**Conclusion:**

The general biology of *V. squamosa *has not changed substantially from that of a typical, nonparasitic *Vespula *wasp. For example, population sizes of the host and its parasite appear to be similar, in contrast to other social parasites, which often display lower population sizes than their hosts. In addition, parasitism has not caused the mating behavior of *V. squamosa *queens to deviate from the high levels of multiple mating that typify *Vespula *wasps. This stands in contrast to some socially parasitic ants, which revert to mating with few males. Overall, the general similarity of the genetic structure of *V. maculifrons *and *V. squamosa *presumably reflects the fact that *V. squamosa *is still capable of independent colony founding and thus reflects an intermediate stage in the evolution of social parasitism.

## Background

The complex, interactive communities displayed by highly social insects represent an important and highly successful evolutionary innovation [1,2]. The success of social insects arises largely from their extraordinary cooperative and helping behaviors [2-5]. However, the intricate social organization displayed by social insects may also be exploited and manipulated. For instance, many social insects are subject to social parasitism [6,7]. Social parasites benefit from brood care or other resources at the expense of the society of a social host species [8].

In hymenopteran social insects (ants, some bees, and some wasps), social parasitism may take several forms [3,7-10]. The most extreme social parasites are completely dependent on their host taxa. Queens of these obligate parasites enter active host colonies, kill the resident queen, and use the remaining worker force of the host to rear their own parasitic offspring. These obligate parasites do not produce their own workers, and are completely reliant on the workers of the host to complete their life cycle. Alternatively, some social parasites display facultatively parasitic behavior. Queens of these species may usurp colonies of their hosts in some cases, but may also reproduce independently under other conditions. These facultative social parasites are of particular interest, because they may represent an intermediate stage in the evolution of socially parasitic behavior [11,12].

**Figure 1:**
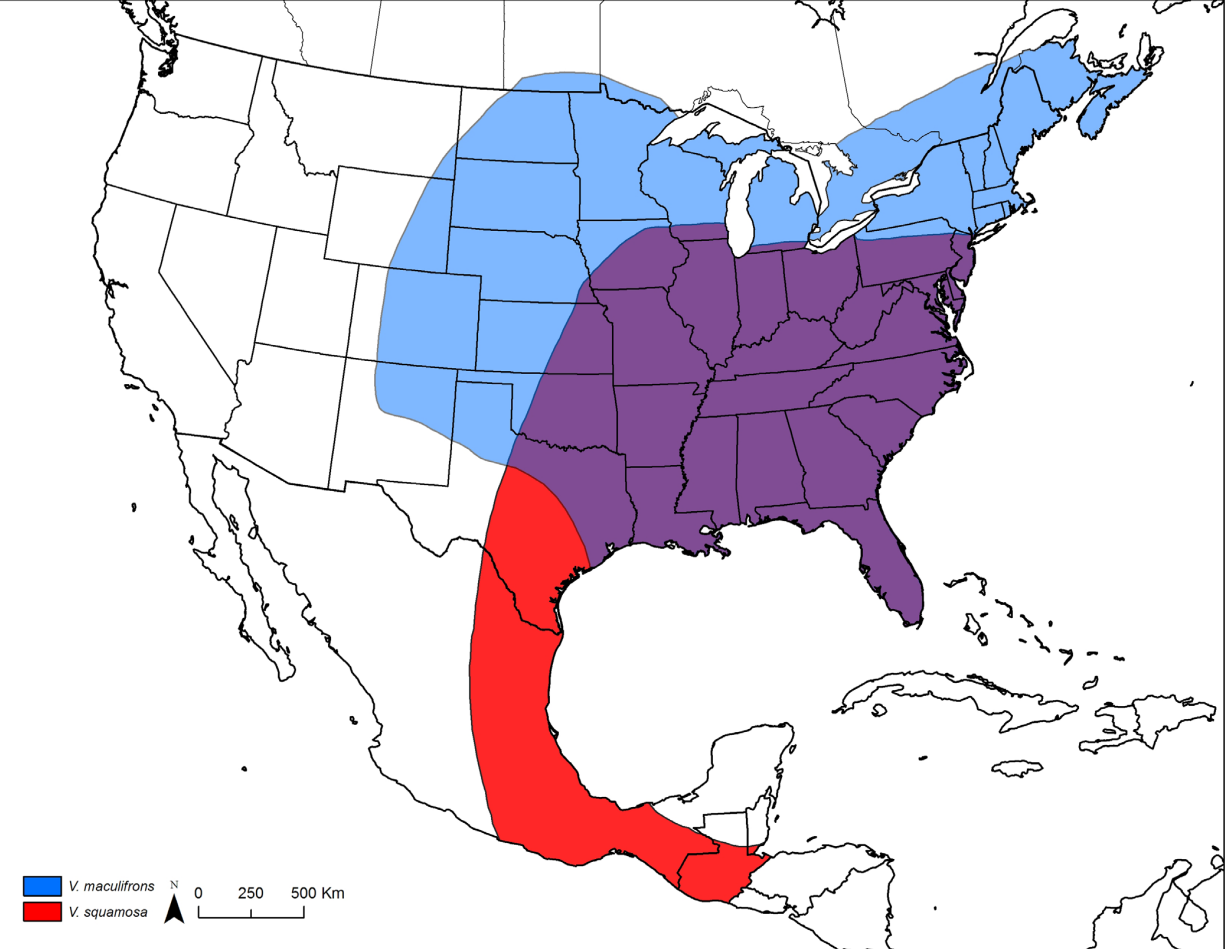
**Distribution of *V. maculifrons *and *V. squamosa *in North America.** The range of the parasite *V. squamosa *largely overlaps with that of the host *V. maculifrons *(adapted from [17]).

The social wasp *Vespula squamosa*, commonly known as the southern yellowjacket, is a facultative social parasite [11,13-15]. *Vespula squamosa *is found throughout the eastern part of the United States extending south to Honduras (Fig. 1) [16,17]. It is thought that *V. squamosa *queens can found colonies independently under some circumstances [11], because no known hosts live in the southern part of the range of *V. squamosa*. However, throughout most of its range, *V. squamosa *is considered to be parasitic [13].

*Vespula squamosa *queens parasitize host taxa by usurping active nests established by other queens [11,12,14,15,18]. *Vespula squamosa *queens are known to primarily parasitize species in their own genus. However, there are reports of *V. squamosa *queens usurping colonies of distantly related taxa, such as the hornet *Vespa crabro *[18]. Where parasitism occurs, *V. squamosa *queens emerge relatively late in the season and seek out already established host colonies to usurp [12-14]. Once host colonies are located, the *V. squamosa *queen kills the resident queen and assumes possession of the colony. The remaining host workers help the *V. squamosa *queen rear her own worker and sexual offspring. Eventually the usurped workers die and the entire colony comes to be inhabited by *V. squamosa *individuals.

In the southeastern part of the United States, the principal host of *V. squamosa *is the eastern yellowjacket, *V. maculifrons *(Fig. 1) [14,15]. In some areas, 40% of *V. maculifrons *colonies fall victim to *V. squamosa *parasitism [19]. Moreover, 80% of *V. squamosa *colonies show clear evidence of having originated by parasitism of *V. maculifrons *colonies in this part of the country. In fact, the frequency of parasitism is likely higher, because usurped nests can only be detected if nest take-over occurs subsequent to significant cell construction by the host [15].

Social parasitism may play an important role in determining the levels of genetic variation and structure of the two interacting taxa [7,20-22]. For example, *V. squamosa *queens represent a significant mortality factor for *V. maculifrons*, owing to the relatively high rate of nest takeover. Nest parasitism may thereby potentially depress the amount of genetic variation in *V. maculifrons *populations by limiting *V. maculifrons *population size. Moreover, the number of *V. squamosa *nesting sites is potentially constrained by the presence of *V. maculifrons *colonies in the population, because *V. squamosa *queens primarily found nests by usurping those already initiated by *V. maculifrons*. Evolutionary interactions of social parasites are also known to be influenced by rates of migration for a parasite and its host [7]. The interaction between migration and local adaptation may lead to a coevolutionary arms race between parasites and their hosts with gene flow as the primary currency [23]. In sum, parasite range and effective population size are related to the degree of population structure of its host. However, the degree that this will influence demography and genetic structure is currently unknown in a facultative social parasite. Thus, our aim was to understand the genetic structure and levels of genetic variation in *V. maculifrons *and *V. squamosa *to determine if the parasitic lifestyle differentially affected gene flow and population size in the two taxa.

The relationship between a social parasite and its host also likely affects the breeding systems of both taxa. For instance, signaling systems between hosts and parasites provide an interesting paradox associated with genetic diversity generated by the breeding system of the host [10]. Increased within-colony genetic diversity of a host species arising as a result of multiple queens within colonies or multiple mating by queens may lead to a superior defense against pathogens or enhanced division-of-labor (e.g., [24-27]). However, high levels of within-colony genetic diversity may also enhance the ability of social parasites to invade host colonies, because it leads to a greater diversity of recognition cues [28,29]. In addition, the breeding system of the parasite may also be affected by the host-parasite interaction. Specifically, Sumner *et al*. [30] recently found that parasitism may affect mate number of the parasitic queen. They discovered that queens of a socially parasitic ant mated singly, whereas queens of the closely-related host species mated multiply. These results suggested that benefits to multiple mating were only accrued in a free-living lifestyle. In contrast, the obligate parasite reverted to single mating, because multiple mating apparently did not provide benefits to the parasitic lifestyle.

In *Vespula *species, within colony genetic diversity is directly related to queen mate number because annual *Vespula *colonies are always headed by a single queen. Indeed, queens of all *Vespula *taxa mate with multiple males [31-37]. We thus aimed to investigate if the host-parasite relationship between *V. squamosa *and *V. maculifrons *altered mate number of the parasite *V. squamosa *relative to its host *V. maculifrons*. In this system, however, because *V. squamosa *colonies exist as free-living entities for most of their life cycle (i.e., after usurpation and colony takeover is complete), we predicted that *V. squamosa *would not have reverted to a mating system with reduced number of mates, as has been found in other obligate social parasites.

Overall, the primary objectives of this study were to compare levels of genetic structure and breeding systems of a social wasp and its social parasite. We expected that the parasitic interaction would lead to differences in the genetic structures, but not the breeding systems, of the two species. We conclude this study by discussing how the observed patterns of genetic variation provide insight into how parasitic interactions affect genetic structure within and between species.

## Methods

### Samples

**Figure 2:**
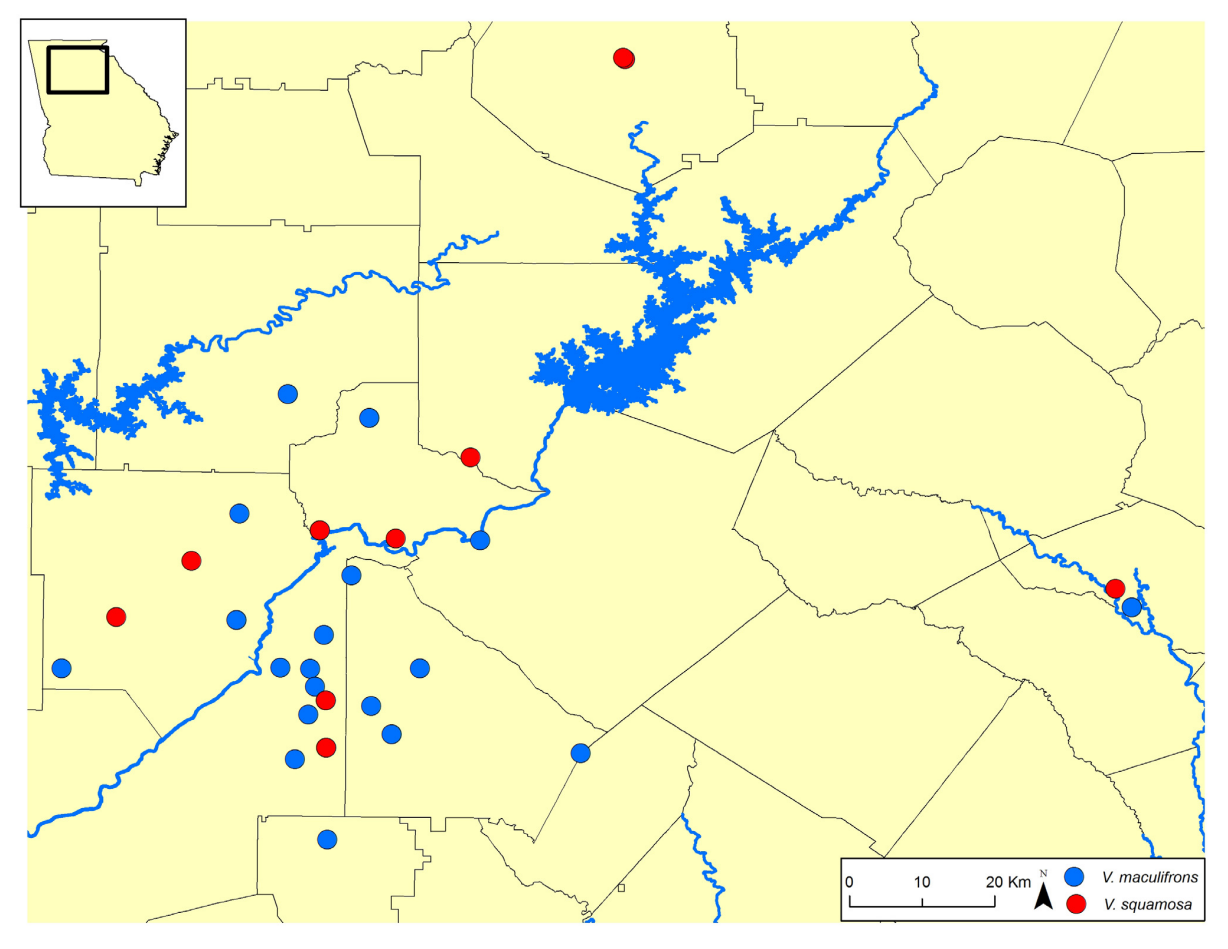
**Locations of 37 *V. maculifrons *and 13 *V. squamosa *colonies collected in this study.** All samples were obtained from the state of Georgia (inset, see also Fig. 1). Lines denote county boundaries within the state. The greatest pairwise distance between colonies exceeds 120 km and colonies of both species were sampled from overlapping regions.

We collected 37 *V. maculifrons *and 13 *V. squamosa *colonies in and around the city of Atlanta, GA, USA (Fig. 2). Colonies were anesthetized with ether, extracted from the ground, and brought back to the lab. Several workers from each colony were then placed in 95% ethanol for subsequent genetic analysis. Colony collection occurred late in the season (September – November), when usurpation would have been complete, so we did not find mixed colonies containing workers of both species. In addition, most colonies had initiated the reproductive phase of their development and were already producing new queens and males.

### Microsatellite loci

We determined the utility of several previously developed microsatellite loci in providing genetic information in *V. maculifrons *and *V. squamosa*. Specifically, we investigated if the loci cloned by Thorèn *et al. *[38] in *V. rufa*, Hasegawa and Takahashi [39] in *Vespa mandarinia*, and Daly *et al. *[40] in *V. vulgaris *were genetically informative in our study taxa. For these assays, we determined if each locus would PCR amplify in *V. maculifrons *and *V. squamosa*. If a locus amplified, we then used agarose gel electrophoresis to determine if the locus displayed size variation in a subset of individuals. In the end, we obtained the genotype of all sampled workers of both species at the eight loci, LIST2003, LIST2004, LIST2013, LIST2019, LIST2020, Rufa 5, VMA-3, and VMA-6, which amplified and were polymorphic in both taxa.

### Estimates of variability

Members of social insect colonies do not represent independent genetic samples because they are related. Consequently, it is necessary to remove the effects of relatedness among colony members to effectively analyze population level data. We avoided the problem of genetic nonindependence of colonymates by inferring the genotypes of the queen and male(s) that produced workers from each colony. Paternity analyses in hymenopteran taxa, such as *Vespula*, are particularly straightforward because males are haploid and full siblings always display the same multilocus haplotype derived from their father. Our analyses thus resulted in a set of diploid (queen) and haploid (male) individuals that were genetically independent. We used the genotypes of these inferred parental individuals to obtain estimates of genetic variability.

Based on the reconstructed male and queen genotypes, we calculated the allelic richness at the eight loci in *V. maculifrons *and *V. squamosa*. In addition, we used the rarefaction method of Petit *et al. *[41] to correct for the difference in the number of genes sampled in the two species. This method estimates the number of alleles that would be observed in an equal sample of genes from multiple groups based on the number of alleles observed in the actual unequal samples obtained. The use of the standardized allele richness allows for comparisons in the diversity of samples of different sizes. We then calculated the effective number of alleles and gene diversity of each locus to obtain general measures of the overall variability for the markers.

### Population structure

To avoid problems caused by nonindependence of workers sampled from the same colony, we used a resampling technique that yielded unbiased measures of population genetic structure [33]. Specifically, we used reduced data sets that included only one individual per colony for population analyses. Briefly, a computer program was written to randomly select a single individual's multilocus genotype from each colony, yielding a new data set with the number of individuals sampled equal to the number of colonies. This procedure was repeated 25 times to produce 25 such data sets. Each of these data sets was then used to calculate the population statistic of interest, and the median of the 25 values was taken as the unbiased estimate. Probability tests implemented by the program GENEPOP 3.4 [42] were used in conjunction with this resampling procedure to determine the significance of genotypic disequilibrium between microsatellite markers and deviations from Hardy-Weinberg equilibrium.

We next determined if *V. maculifrons *or *V. squamosa *populations showed evidence of isolation by distance over the geographical scale considered. We first calculated estimates of *F*_ST _between all pairs of colonies for each species using GENEPOP. Thus, colonies, which consisted of groups of sampled workers, were considered as 'populations' for these analyses. Significance of the correlation between geographical and genetic distance was assessed with a Mantel test. The relationship was deemed to be significant if the observed correlation was greater than 5% of the 10,000 randomly generated values. Spearman's rank order correlation coefficient (*r*_S_) was used to quantify the association between the genetic distances between nests and the geographical distances that separated them.

### Mating system

We used the molecular genetic data derived from the worker genotypes to investigate how the breeding systems of *V. maculifrons *and *V. squamosa *differed. Because annual *Vespula *colonies are headed by single queens, within colony genetic diversity provides insight into the queen's mating history. We thus used the genetic data to determine the number of males to which queens were mated. We then compared mate number in the two taxa via a Kruskal-Wallis test. We next calculated the effective paternity (*k*_*e*3_) for workers using the method of Nielsen *et al. *[43]. The metric *k*_*e*3 _includes information on the number of times a queen mates and the unequal contributions of a queen's male mates to offspring. We also tested if the estimates of *k*_*e*3 _obtained for *V. maculifrons *and *V. squamosa *differed using a Kruskal-Wallis test.

Additionally, we investigated if the distribution of mate number in the two species matched known distributions, as might be expected if particular biological processes (such as constant mate search time or equal probability of mating with particular numbers of males) affected the mating behavior of queens [44]. Specifically, we fitted a Gaussian distribution to queen mate number. We expected that mate number would match this distribution if number of mates were a random function of time and mating opportunity. In contrast, deviations of mate number from the Gaussian distribution might indicate the action of selection operating in these species.

We calculated the magnitude of reproductive skew of males mated to queens using the metric *B *of Nonacs [45]. The 95% confidence intervals for each estimate of *B *were estimated to determine the significance of skew. Values of *B *were considered to be significant if the 95% confidence intervals failed to overlap 0. Both the skew and significance of skew were calculated using the program Skew Calculator [45]. As was the case with our estimates of *k*_*e*3_, we tested if estimates of *B *differed between species via a Kruskal-Wallis test.

We calculated the relatedness of workers belonging to the same colony using the program RELATEDNESS [46]. We then estimated the relatedness of the queens to their male mates and the relatedness of males mated to single queens to determine if inbreeding occurred or if related males mated the same queen. Standard errors for all estimates were obtained by jackknifing over loci. Finally, we compared our relatedness estimates with similar data collected over two decades ago to determine how the parasitic relationship may have changed mating structure over time.

## Results

### Samples

We sampled 40.56 ± 23.51 (mean ± SD) and 45.62 ± 8.31 workers from each of 37 *V. maculifrons *and 13 *V. squamosa *colonies, respectively (Fig. 2). Our total sample size thus consisted of 1501 and 463 workers from the two taxa. The maximum distance between colonies in this study spanned approximately 120 km. Moreover, the sampling locations of *V. maculifrons *showed considerable overlap with those of *V. squamosa *(Fig. 2).

### Microsatellite loci

We determined if the 43 microsatellite loci developed in related species within the Vespidae were informative in *V. maculifrons *or *V. squamosa*. Overall, we found that 16 loci amplified and were variable in both species (Appendix). We used eight of these 16 loci for our study (see Methods). We analyzed reduced data sets consisting of only one individual per colony to determine if loci were in Hardy-Weinberg equilibrium in the two species. We found no evidence of disequilibrium among workers in either *Vespula *taxon (*P *> 0.1 for all loci in both species; overall combined *P *= 0.2388 in *V. maculifrons *and *P *= 0.5304 in *V. squamosa*). In addition, there was no evidence of linkage disequilibrium between any pair of loci (*P *> 0.3 for all pairs of loci in both species). The eight loci that we ultimately utilized for this study showed substantial variability in the two taxa, with gene diversities (*h*) often exceeding 0.7 (Table 1). The exceptions were the loci Rufa-5 in *V. maculifrons *and LIST2019 in *V. squamosa*, where the variability was somewhat less. Regardless, the variability of the loci was high enough such that the probability of any two males having the same multilocus genotype (nondetection error) was extremely low (<< 0.0001).

**Table 1:** Variability metrics of microsatellite loci in *V. maculifrons *(*Vmac*) and *V. squamosa *(*Vsqu*).

	*N*^a^		*A*_*obs*_^b^		*A*_50_^c^		*A*_*eff*_^d^		*h*^e^		Range (bp)^f^	
Locus	*Vmac*	*Vsqu*	*Vmac*	*Vsqu*	*Vmac*	*Vsqu*	*Vmac*	*Vsqu*	*Vmac*	*Vsqu*	*Vmac*	*Vsqu*
LIST2003	259	107	31	17	20.319	14.865	17.642	8.375	0.943	0.889	185–224	173–204
LIST2004	253	113	20	8	13.073	6.623	8.691	4.155	0.885	0.766	147–181	119–149
LIST2013	256	107	20	18	11.839	13.911	7.241	6.357	0.862	0.850	184–213	173–201
LIST2019	224	108	11	4	8.373	3.711	3.907	1.534	0.744	0.352	126–152	122–132
LIST2020	256	110	19	19	12.276	15.061	7.360	10.168	0.864	0.910	226–268	322–356
Rufa-5	213	107	3	11	2.223	9.066	1.206	5.011	0.171	0.808	136–140	153–175
VMA-3	219	77	16	14	12.543	13.389	9.556	10.420	0.895	0.916	260–286	254–282
VMA-6	256	109	39	20	22.071	15.329	17.722	10.287	0.944	0.911	262–323	267–295

^a^Number of genes sampled obtained from the inferred genotypes of females and males. Unequal sample sizes among loci within each species result from missing or incomplete data.

^b^Total number of observed alleles.

^c^Number of alleles that would have been observed if only 50 genes had been sampled.

^d^Effective number of alleles.

^e^Expected heterozygosity.

^f^Range in allele size.

### Estimates of variability

The standardized number of alleles (*A*_50_, defined as the number of alleles expected to be observed if only 50 genes had been sampled from a given population; Table 1; [41]) did not differ significantly between the taxa [12.8 ± 6.3 (mean ± SD) for *V. maculifrons *and 11.5 ± 4.45 for *V. squamosa*; paired *t*-test, *t *= 0.74, *P *= 0.48] suggesting that genetic variability was not substantially different between the parasite and its host. In addition, the effective number of alleles did not show strong differences between species (9.2 ± 5.90 for *V. maculifrons *and 7.0 ± 3.31 for *V. squamosa*; paired *t*-test, *t *= 1.27, *P *= 0.24). Moreover, the variability at particular loci was similar in the two species. Specifically, the estimates of *A*_50 _and expected heterozygosity (*h*) were significantly correlated across loci in the two taxa (Spearman's correlation coefficient; *A*_50_, *r*_S _= 0.738, *P *= 0.0366; *h*, *r*_S _= 0.762, *P *= 0.0280).

### Population structure

**Figure 3:**
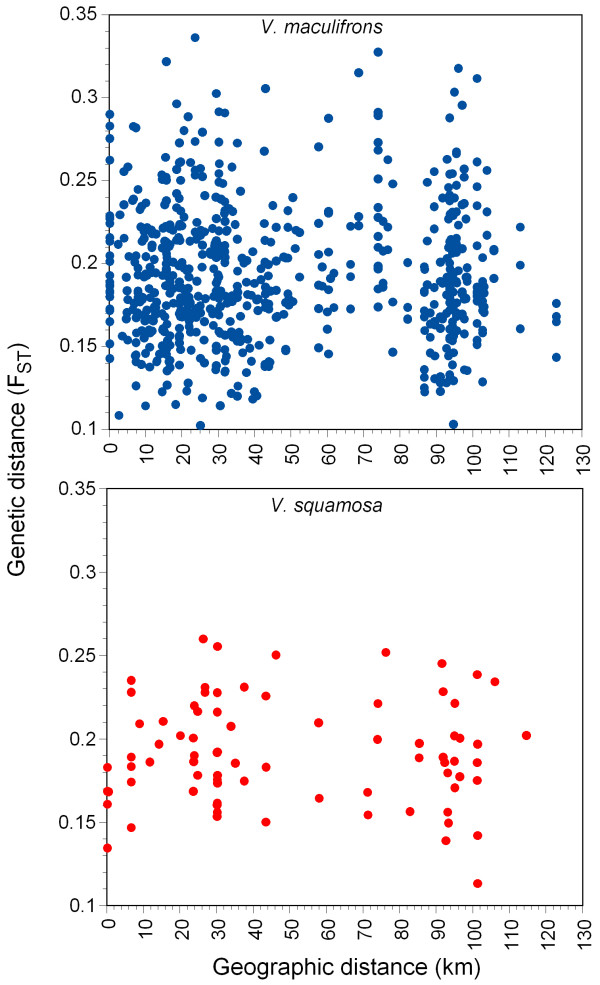
**Relationship between genetic differentiation and geographic distance of workers sampled from *Vespula *colonies.** There was no evidence for genetic isolation by distance in these species over this range.

The overall estimates of genetic differentiation between colonies in *V. maculifrons *and *V. squamosa *were *F*_ST _= 0.1953 and 0.1914, respectively. We found no evidence of genetic isolation by distance among colonies in either species (Fig. 3). The correlation between geographic distance and genetic distance was low and nonsignificant in *V. maculifrons *(*F*_ST_, *r*_S _= 0.0176, *P *= 0.4694) and *V. squamosa *(*F*_ST_, *r*_S _= 0.0036, *P *= 0.4748). Thus, both of these *Vespula *species are apparently able to mate at random over the range in which sampling occurred.

### Mating system

**Figure 4:**
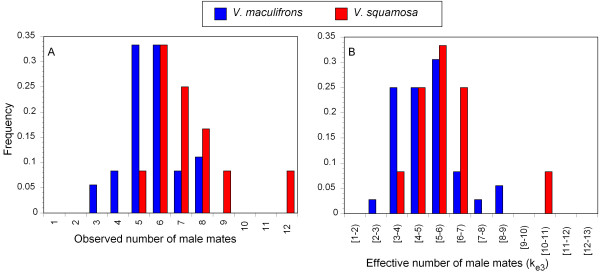
**Distribution of (A) observed mate number and (B) effective mate number (*k*_*e*3_) for *V. maculifrons *and *V. squamosa *queens.** Within each species the distribution effective mate number is reduced relative to observed mate number because of unequal sperm use by queens. The distributions of observed mate number differed significantly from a Gaussian distribution in both species due to an excess of queens mated to intermediate numbers of males.

As expected, we found that the genotype distributions of workers within colonies always were consistent with the presence of a single queen mated to multiple males. *V. maculifrons *queens mated with 5.64 ± 1.27 males (mean ± SD; range of 3 – 8, Fig. 4), whereas *V. squamosa *queens mated with 7.25 ± 1.86 males (range of 5 – 12; Fig. 4). The mate number of queens in the two taxa differed significantly from each other (Kruskal Wallis *S *= 412.5, *P *= 0.0037). The estimates of effective mate number (*k*_*e*3_) in *V. maculifrons *and *V. squamosa *were 4.96 ± 1.40 and 5.58 ± 1.66, respectively (range in *k*_*e*3 _of 2.61 – 8.82 in *V. maculifrons *and 3.75 – 10.15 in *V. squamosa*; Fig. 4). In contrast to the actual mate number, the estimates of effective mate number did not differ significantly between species (*S *= 343, *P *= 0.2482).

We then turned our attention to the reproductive skew of males mated to the same queen. The mean magnitude of skew was *B *= 0.0273 ± 0.0409 in *V. maculifrons *and *B *= 0.0433 ± 0.0411 in *V. squamosa*. These values were not significantly different from each other (S = 371, *P *= 0.0685). However, the estimates of skew were significantly greater than zero in only 13 of the 36 *V. maculifrons *colonies, whereas the estimates of skew were significant in 10 of the 12 *V. squamosa *colonies. Thus, the proportions of colonies in which the skew was significant in the two taxa differed (*G*_1 _= 8.553, *P *= 0.0034). Nevertheless, although paternity skew was statistically significant in many colonies, the overall magnitude of *B *was low and the actual mate numbers were similar to the effective mate numbers in both species.

We investigated if the distribution of queen mate number in the two species fit known distributions to determine if simple biological processes could explain patterns of queen mating behavior [44]. We found that the distribution of mate number in *V. maculifrons *and *V. squamosa *differed significantly from that expected under a Gaussian distribution (Shapiro-Wilk test; *V. maculifrons*, *W *= 0.920, *P *= 0.0125; *V. squamosa*, *W *= 0.852, *P *= 0.039). The failure of these distributions to fit the data arose from an observed excess of queens that mated with an intermediate number of males (Fig. 4).

Nestmate *V. maculifrons *and *V. squamosa *workers had relatedness values significantly greater than zero (Table 2). However, the relatedness of queens to their male mates did not differ significantly from zero (Table 2), as judged by the fact that the 95% confidence intervals (*r *± 1.96 * SEM) overlapped zero. This indicated that inbreeding does not usually occur in either of these species. Furthermore, we found that relatedness of *V. maculifrons *males mated to single queens was not significantly different from zero (Table 2). But we did find weak evidence that relatedness among *V. squamosa *males mated to single females was significantly greater than zero. Much of this signal, however, was due to a single colony in which the relatedness among male mates was surprisingly high (*r *= 0.312).

**Table 2:** Relatedness (± SEM) estimates for *V. maculifrons *and *V. squamosa*.

Relatedness	V. maculifrons	V. squamosa
Nestmate workers	0.373 ± 0.009 ***	0.357 ± 0.019 ***
Queens to their male mates	0.014 ± 0.010	0.016 ± 0.020
Among males mated to single queens	0.023 ± 0.014	0.053 ± 0.024 *

Estimate differs significantly from zero at the 0.05 (*) or 0.001 (***) level.

## Discussion

The interaction between a parasite and its host may lead to an evolutionary arms race between the taxa. Microparasites often have large population sizes and fast generation times, and may be able to win the evolutionary arms race with their hosts [47]. In contrast, social parasites may have reduced effective population sizes owing to their dependence on host colonies and the 1:1 ratio of parasite to host colony replacement. Moreover, the difference between the level of local adaptation between a host and its parasite is determined by the rates of gene flow within host and parasite populations [23]. Limited gene flow may lead to local coadaptation between hosts and parasites [23,48,49]. In contrast, theoretical models predict that increasing rates of gene flow among host populations can influence parasite population demography such that population densities are reduced, potentially leading to the extinction of the parasite [7,50].

This study represents one of the first joint analyses of levels of genetic variation and breeding system in a parasitic social wasp and its primary host. The main focus of this study was to understand if the parasite *V. squamosa *displayed significant differences in its mating system, levels of genetic variation, and population genetic structure from its host *V. maculifrons*. Subsequently, our investigation led us to more closely compare and contrast the mating system of the two species in order to understand what factors might affect mating behavior. Overall, we found strong similarities in the genetic structure and mating system of the host, *V. maculifrons*, and its parasite, *V. squamosa*. In addition, our investigation of mating biology uncovered patterns consistent with the effects of selection operating on mate number in both taxa.

### Population genetic variation and structure

One of the more striking results arising from our investigation of levels of genetic variation was that measures of allelic diversity were not appreciably different between *V. squamosa *and *V. maculifrons*. Population genetics theory suggests that the amount of genetic variation maintained within a population is related to the effective population size [51]. Therefore, the shared patterns of diversity among these species suggest that long-term effective population size is not substantially different in the two taxa.

Furthermore, we failed to find evidence for genetic isolation by distance in either *Vespula *species. The absence of isolation by distance within both taxa suggests that these *Vespula *wasps exhibit substantial levels of gene flow within the sampling range of this study. This finding is consistent with the high flight capacity of *Vespula *wasps, which are naturally capable of dispersing over considerable distances [52,53]. In addition, hibernating *Vespula *queens may readily be moved via accidental human transportation thereby increasing their effective migratory abilities [54,55].

However, our finding of a lack of geographic structure in *V. maculifrons *and *V. squamosa *differs somewhat from that found in a previous study of the congener *V. germanica *in Australia [33]. In that investigation, *V. germanica *was found to display genetic isolation by distance on approximately the same scale investigated in this study. We note, however, that *V. germanica *is a recent invader to Australia [56]. Thus, the population dynamics of *V. germanica *in Australia likely differ from those of *V. maculifrons *and *V. squamosa *in their native range in the United States. In particular, the observed isolation by distance in *V. germanica *populations in Australia may reflect non-equilibrium conditions that will ultimately fade as gene flow swamps local genetic differentiation [17].

The overall similarity of levels of genetic variation and low levels of structure in *V. maculifrons *and *V. squamosa *stand in contrast to expectations derived from other studies of social hymenopteran parasites. For example, ant social parasites often have low population sizes and dispersal capabilities [7]. In accord with these predictions, Trontti et al. [57] found that an inquiline parasite species exhibited much greater genetic substructuring than was found in its host. Brandt et al. [58] also found substantial genetic structure in two species of parasitic ants, although the magnitude of structuring did not differ substantially from that of the host species. Thus our findings indicate that social parasites need not show levels of variation distinctly different from their hosts.

### Queen mate number and mating behavior

We compared the mating system of *V. maculifrons *and *V. squamosa *in order to investigate if there were differences between the two species that might be associated with parasitic behaviors. As expected, we found that all colonies of both species were headed by only a single queen, and that all queens of both species were polyandrous [35-37]. Our estimates of worker relatedness for *V. maculifrons *and *V. squamosa *(0.373 and 0.357, respectively; Table 2) did not differ significantly from earlier estimates obtained for these species (0.320 and 0.403; [35]). Thus, we found no evidence for temporal variation in the mating systems of these two taxa from samples obtained some 23 years apart. Although only tangential to this study, this result is noteworthy. Few studies have investigated temporal stability of mating structure. Here, we found that structure did not change over a 23 generation time-span. This stability in general mating structure suggests that selective factors affecting mating behavior have remained constant over that time.

We also did not find any convincing evidence of inbreeding between queens and males of both the parasite and its host. In addition, the relatedness of males mated to the same queen tended to be low in both taxa (Table 2). These data are consistent with our understanding of the mating system of *Vespula *wasps. Namely, although inbreeding is possible under laboratory conditions [59], matings in natural settings tend to take place away from the nest and result in outbreeding [18,32,60]. Thus, the parasitic behavior of *V. squamosa *has not led to a cycle of inbreeding as occurs in some socially parasitic ants [7,61].

Our primary interest in studying queen mate number was to test hypotheses related to how mate number should evolve in social parasites. We predicted that mate number would be similar in the two taxa, because *V. squamosa *colonies exist as free-living entities for a substantial part of their life cycle. Therefore, they should retain benefits of multiple mating [37], such as superior task performance of parasite defense associated with increased within-colony genetic diversity, unlike obligate social parasites that are more intimately associated with their hosts [30]. Interestingly, we found that queen mate number differed significantly between the species, with parasitic queens tending to have more mates than host queens. This finding may result from a regime of strong selection of parasites on hosts. In this case, hosts of social parasites might be selected to develop less genetically diverse colonies to increase colony uniformity [7] and hence increase parasite recognition. However, this is not likely to be the case in this system, as levels of within-colony genetic diversity, the currency of multiple mating, did not differ substantially between the species. Thus, overall, our results suggest that *V. squamosa *does indeed bear the benefits of multiple mating likely as a result of the free-living portion of its life cycle [30].

We also found that effective mating frequency of queens was similar between the species. This suggests that unequal use of sperm reduces the effective mate numbers to approximately equal levels in both species, despite the fact that actual queen mate number differed significantly in the two taxa. Although the magnitude of paternity skew was similar between species, such skew was significant more frequently in *V. squamosa*. Male skew has important implications for the reproductive success of males and, potentially, for how colony members allocate resources to the production of sexuals [62]. The underlying mechanisms resulting in the variation of male reproductive skew between the two species remain unclear at this time, but may result from female choice of male sperm or male-male competition occurring among sperm within the female reproductive tract.

Finally, one striking feature of our data was the similarity in the shape of the distribution of mate number in both species. In particular, the distribution of the number of male mates (Fig. 4) indicated that the extremes at both ends of the distribution had been somewhat truncated. This suggests that the number of mates observed does not simply result from random interactions between queens and males. One explanation for the observed pattern is that mate number is under stabilizing selection. Specifically, mating to too few males may be maladaptive [63], whereas mating to too many males may be costly [64]. In fact, Goodisman *et al*. [37] identified a positive correlation between a fitness correlate and number of matings by queens in *V. maculifrons*, indicating that there is a selective advantage to polyandry. Regardless, the patterns of mating behavior found here suggest that similar factors may influence queen mate choice decisions in *V. squamosa*. However, queens in each species may achieve the optimal effective mate number via different evolutionary strategies. That is, *V. squamosa *queens mate with more males, but have greater skew in sperm usage, while *V. maculifrons *queens mate with fewer males, but have less skew.

## Conclusion

The overall similarity in population genetic makeup of *V. maculifrons *and *V. squamosa *may reflect the fact that *V. squamosa *represents an intermediate stage in the evolution of social parasitism. Taylor [12] proposed that parasitism in *Vespula *develops through four stages. First, queens display intraspecific, facultative, temporary social parasitism, whereby conspecific queenless nests are taken over by queens searching for a nesting site. Second, a species may evolve interspecific, facultative, temporary social parasitism. In this case, a queen searching for a nest occasionally takes over the queenless nest of a heterospecific host. Third, a species may evolve to interspecific, obligatory, temporary parasitism. In this third evolutionary step, the parasitic queen loses her ability to found new colonies but still produces her own workers once she takes over an established heterospecific nest. And finally, a species may evolve to display interspecific, obligatory, permanent parasitism, where the parasitic worker caste is completely lost.

Extreme specialists (obligate, workerless parasites) are usually rare, restricted locally, and highly genetically structured. However, the genetic fingerprint of *V. squamosa *may be somewhat less defined by its parasitic life style because it falls somewhere between stages two and three in Taylor's proposed series of the evolution of parasitism. In support of this hypothesis, Hölldobler and Wilson [3] point out that facultatively parasitic ants, or those that are more primitively parasitic, tend to be widely distributed, as seems to be the case with *V. squamosa*.

Future research in the study of the social parasite *V. squamosa *should aim to determine the extent that this species is facultatively versus obligately parasitic. In particular, a study of geographic variation in parasitic behavior and frequency would be informative. Additionally, it has been suggested that social parasites that exploit multiple hosts may form host races. A previous study failed to find evidence for such host race formation in *Polistes *[65]. However, closer study of *V. squamosa*, which parasitizes multiple hosts [18], would provide another system in which to study how parasitism can lead to increased biodiversity of social insects.

## Authors' contributions

EAH, JLK, and MADG conceived the study and participated in its design and coordination. EAH and JLK carried out laboratory analyses. EAH and MADG analyzed the data and drafted the manuscript. All authors read and approved the final manuscript.

## Appendix

Utility of 43 microsatellite loci in V. maculifrons and V. squamosaa (Table 3)

**Table 3:** Utility of 43 microsatellite loci in *V. maculifrons *and *V. squamosa*^a^.

Locus	*V. maculifrons*	*V. squamosa*
		
LIST2001	Var	-
LIST2002	Var	-
LIST2003	Var	Var
LIST2004	Var	Var
LIST2006	Var	+++
LIST2007	Var	Var
LIST2008	Var	Var
LIST2009	+++	+++
LIST2010	Var	+++
LIST2011	-	-
LIST2012	-	-
LIST2013	Var	Var
LIST2014	-	-
LIST2015	Var	Var
LIST2016	Var	Var
LIST2017	Var	-
LIST2018	-	-
LIST2019	Var	Var
LIST2020	Var	Var
		
Rufa 1	-	-
Rufa 2	Var	Var
Rufa 3	+++	Var
Rufa 4	-	-
Rufa 5	Var	Var
Rufa 6	-	-
Rufa 7	+++	+++
Rufa 8	-	-
Rufa 9	-	Var
Rufa 10	-	-
Rufa 11	Var	+++
Rufa 12	Var	Var
Rufa 13	-	Var
Rufa 14	-	+++
Rufa 15	Var	Var
Rufa 16	-	-
Rufa 17	+++	Var
Rufa 18	Var	Var
Rufa 19	Var	+++
		
VMA-3	Var	Var
VMA-4	-	-
VMA-6	Var	Var
VMA 7	+++	+++
VMA-8	Var	-

^a^Designations indicate that a particular locus failed to PCR-amplify (-), PCR-amplified but was not variable (+++), or both PCR-amplified and was variable (VAR). Variability was assessed from visualization of alleles via agarose gel electrophoresis.
